# Age-Specific Risk Factors for Advanced Stage Colorectal Cancer, 1981–2013

**DOI:** 10.5888/pcd15.170274

**Published:** 2018-08-23

**Authors:** Kevin J. Moore, Daniel A. Sussman, Tulay Koru-Sengul

**Affiliations:** 1 Department of Public Health Sciences, University of Miami School of Medicine, Miami, Florida; 2 Department of Medicine, University of Miami School of Medicine, Miami, Florida; 3 Sylvester Comprehensive Cancer Center, University of Miami School of Medicine, Miami, Florida

## Abstract

**Introduction:**

Epidemiologic studies have identified an increase in colorectal cancer (CRC) among younger adults. By using a statewide population-based cancer registry, this study examines sociodemographic and clinical disparities in CRC and characterizes advanced stage CRC risk factors with specific attention to age-specific risk factors.

**Methods:**

Data from the Florida Cancer Data System from 1981 through 2013 were analyzed for adult CRC patients. Patients were divided into 2 age groups: younger than 50 years and 50 years or older. Stage of presentation was categorized as early (localized) or advanced (regional or distant). Multivariable logistic regression models adjusted for sociodemographic and clinical characteristics were fitted to identify risk factors for advanced stage CRC presentation. Adjusted odds ratios were calculated with 95% confidence intervals.

**Results:**

From 1981 through 2013, there were 182,095 Florida adults diagnosed with CRC. Those aged younger than 50 years were significantly more likely to have advanced stage CRC compared with those aged 50 or older. Among those younger than 50 years, current and former tobacco smokers and those of black or other race were significantly more likely to have advanced stage CRC. Among those aged 50 or older, Hispanics had significantly higher risk of advanced stage presentation compared with non-Hispanics, although this association was not significant in those younger than 50 years.

**Conclusion:**

We identified significant age-specific risk factors for advanced stage CRC presentation. With CRC incidence on the rise among younger adults, it is important to identify and to target screening and interventions for groups at high risk for advanced stage CRC presentation.

**MEDSCAPE CME**

Medscape, LLC is pleased to provide online continuing medical education (CME) for this journal article, allowing clinicians the opportunity to earn CME credit.

In support of improving patient care, this activity has been planned and implemented by Medscape, LLC and *Preventing Chronic Disease*. Medscape, LLC is jointly accredited by the Accreditation Council for Continuing Medical Education (ACCME), the Accreditation Council for Pharmacy Education (ACPE), and the American Nurses Credentialing Center (ANCC), to provide continuing education for the healthcare team.

Medscape, LLC designates this Journal-based CME activity for a maximum of 1.00 *AMA PRA Category 1 Credit(s)™*. Physicians should claim only the credit commensurate with the extent of their participation in the activity.

All other clinicians completing this activity will be issued a certificate of participation. To participate in this journal CME activity: (1) review the learning objectives and author disclosures; (2) study the education content; (3) take the post-test with a 75% minimum passing score and complete the evaluation at http://www.medscape.org/journal/pcd; (4) view/print certificate.

**Release date: August 23, 2018; Expiration date: August 23, 2019**

**Learning Objectives**

Upon completion of this activity, participants will be able to:

1. Assess the epidemiology of colorectal cancer in the United States
2. Analyze the prevalence of colorectal cancer among adults younger than 50 years
3. Evaluate risk factors for advanced colorectal cancer

**EDITOR**

Caran Wilbanks

Editor, Preventing Chronic Disease

Disclosure: Caran Wilbanks has disclosed the following relevant financial relationships:

Employed by a commercial interest: Partner is employed by Allscripts, Inc.

**CME AUTHOR**

Charles P. Vega, MD, FAAFP

Health Sciences Clinical Professor, University of California, Irvine, Department of Family Medicine; Associate Dean for Diversity and Inclusion, University of California, Irvine, School of Medicine; Executive Director, University of California, Irvine, Program in Medical Education for the Latino Community, Irvine, California

Disclosure: Charles P. Vega, MD, FAAFP, has disclosed the following relevant financial relationships:

Served as an advisor or consultant for: Johnson and Johnson Healthcare

Served as a speaker or a member of a speakers bureau for: Shire Pharmaceuticals

**AUTHORS**

Kevin J. Moore, MD, MPH

Department of Public Health Sciences, Medical Education MD/MPH program, University of Miami Miller School of Medicine, Miami, FL

Disclosure: Kevin J. Moore, MD, MPH, has disclosed no relevant financial relationships.

Daniel A. Sussman, MD, MSPH

Department of Medicine, Sylvester Comprehensive Cancer Center, University of Miami Miller School of Medicine, Miami, FL

Disclosure: Daniel A. Sussman, MD, MSPH, PhD, has disclosed no relevant financial relationships.

Tulay Koru-Sengul, PhD, MHS

Department of Public Health Sciences, Sylvester Comprehensive Cancer Center, University of Miami Miller School of Medicine, Miami, FL

Disclosure: Tulay Koru-Sengul, PhD, MHS, has disclosed no relevant financial relationships.

## Introduction

Despite a decrease in colorectal cancer (CRC) among all adults, recent epidemiologic studies have identified an increase in incidence among adults younger than 50 years of age (1). CRC is among the most common cancers diagnosed in the United States for adults younger than 50 years of age (2). Among all US adults, CRC is the third most commonly diagnosed cancer in both men and women (3). In 2017 in the United States, an estimated 135,430 people were predicted to be diagnosed with CRC and about 50,260 people were predicted to die from the disease (4). In Florida, the 2014 age-adjusted incidence and mortality rates were 35.4 (95% confidence interval [CI], 34.6–36.1) and 13.0 (95% CI, 12.6–13.5), respectively (5). Florida includes specific counties with some of the highest CRC mortality rates nationally (6).

Advanced stage presentation and diagnosis of CRC portends significantly worse survival outcomes for both younger and older adults (7). By using a statewide population-based cancer registry, this study examines sociodemographic and clinical disparities in CRC cases among adults and characterizes advanced stage CRC diagnosis risk factors with specific attention to age-specific risk factors.

## Methods

Data from the Florida Cancer Data System (FCDS) with the linkage to the 2000 US census were analyzed for patients with primary CRC who were aged 18 years or older for 1981 through 2013. Patients who had missing values for age, race, ethnicity, neighborhood socioeconomic status (SES), and Surveillance, Epidemiology, and End Results (SEER) Program tumor stage at presentation were excluded from the analysis.

Sex (female, male), race (white, black, others), ethnicity (Hispanic, non-Hispanic), and smoking status (never, current, former, unknown) were self-reported. The FCDS registry does not contain information regarding patient SES at the time of cancer diagnosis. To be able to incorporate SES into the statistical analysis, we used data from the 2000 US Census to provide a proxy for individual SES. Patients’ US census tract at the time of cancer diagnosis is used for the linkage. Patients’ SES at the time of diagnosis were approximated by neighborhood level SES, which is determined by the patient’s US census tract as the percentage of people living below poverty level as low (20% to 100%), middle-low (10% to <20%), middle-high (5% to <10%), and highest (0% to <5%). Age at diagnosis was divided into younger than 50 years or 50 years or older to align with the starting age for CRC screening recommended by the US Preventive Services Task Force (8). Anatomic sites were colon (ascending, transverse, descending, sigmoid), flexure (hepatic, splenic), rectosigmoid junction, and rectum. Individuals who did not have a specified primary anatomic site in the colon or rectum (ie, colon, not otherwise specified) were also excluded from the analysis. The primary clinical outcome, SEER tumor stage at diagnosis, was categorized as early (localized) or advanced (regional or distant) as a dichotomous variable.

Multivariable logistic regression models for all patients and by age group were fitted to identify risk factors for advanced stage CRC presentation. Adjusted odds ratios (AORs) and 95% CIs were calculated and adjusted for sociodemographic and clinical characteristics for the whole sample as well as by age group. All statistical analyses were completed by using SAS v.9.4 (SAS Institute, Inc). The study was approved by the Florida Department of Health (DOH) and University of Miami institutional review boards.

## Results

In total, 277,562 adults were diagnosed with primary CRC during 1981 through 2013. There were 182,095 adults who satisfied the inclusion and exclusion criteria. Most were aged 50 years or older (93.0%) (Table 1). Approximately 60% of all cases were diagnosed at advanced stage (42.7% regional, 18.9% distant), and among those younger than 50 years, 69.6% were diagnosed at the advanced stage (44.7% regional, 25.4% distant). In patients younger than 50 years with advanced stage presentation, most were men (52.6%), white (78.9%), and non-Hispanic (84.6%). Among patients younger than 50 years, the mean age of diagnosis was 42.5 years (95% CI, 42.4–42.6), while the mean age of diagnosis for those 50 or older was 71.7 (95% CI, 71.7–71.8). Patients younger than 50 years had a greater proportion of current smokers compared with those 50 or older. The most common anatomic site for advanced stage presentation was sigmoid colon for both the younger (27.8%) and older (27.2%) age groups.

**Table 1:** Sociodemographic Characteristics of Colon Cancer Patients, by Age Group, Florida Cancer Data System, 1981–2013

Characteristic	All, n (%)^b^	Age Group and Stage Presentation^a^			
		<50 years		≥ 50 years	
		Early, n (%)^b^	Advanced, n (%)^b^	Early, n (%)^b^	Advanced, n (%)^b^
**Total**	182,095 (100.0)	3,861 (2.1)	8,850 (4.9)	67,380 (37.0)	102,004 (56.0)
**Sex**					
Male	95,502 (52.4)	1,934 (50.1)	4,655 (52.6)	35,612 (52.9)	53,301 (52.3)
Female	86,593 (47.6)	1,927 (49.9)	4,195 (47.4)	31,768 (47.1)	48,703 (47.7)
**Race**					
White	164,865 (90.5)	3,165 (82.0)	6,979 (78.9)	62,112 (92.1)	92,609 (90.8)
Black	15,342 (8.4)	614 (15.9)	1,626 (18.4)	4,688 (7.0)	8,414 (8.2)
Other^c^	1,888 (1.0)	82 (2.1)	245 (2.8)	580 (0.9)	981 (1.0)
**Ethnicity**					
Non-Hispanic	162,633 (89.3)	3,303 (85.5)	7,488 (84.6)	60,687 (90.1)	91,155 (89.4)
Hispanic	19,462 (10.7)	558 (14.5)	1,362 (15.4)	6,693 (9.9)	10,849 (10.6)
**Neighborhood socioeconomic status^d^**					
Lowest	26,705 (14.7)	628 (16.3)	1,596 (18.0)	9,135 (13.6)	15,346 (15.0)
Middle-low	58,224 (32.0)	1,227 (31.8)	2,919 (33.0)	21,162 (31.4)	32,916 (32.3)
Middle-high	65,437 (35.9)	1,236 (32.0)	2,791 (31.5)	25,139 (37.3)	36,271 (35.6)
Highest	31,729 (17.4)	770 (19.9)	1,544 (17.4)	11,944 (17.7)	17,471 (17.1)
**Smoking status**					
Never	82,134 (45.1)	1,890 (49.0)	4,210 (47.6)	30,406 (45.1)	45,628 (44.7)
Current	21,709 (11.9)	702 (18.2)	1,825 (20.6)	6,759 (10.0)	12,423 (12.2)
Former	41,643 (22.9)	446 (11.6)	1,046 (11.8)	15,862 (23.5)	24,289 (23.8)
Unknown^e^	36,609 (20.1)	823 (21.3)	1,769 (20.0)	14,353 (21.3)	19,664 (19.3)
**Anatomic site of cancer**					
Ascending colon	32,860 (18.0)	333 (8.6)	1,054 (11.9)	12,001 (17.8)	19,472 (19.1)
Hepatic flexure	8,682 (4.8)	104 (2.7)	285 (3.2)	3,003 (4.5)	5,290 (5.2)
Transverse colon	15,335 (8.4)	184 (4.8)	656 (7.4)	5,072 (7.5)	9,423 (9.2)
Splenic flexure	6,291 (3.5)	90 (2.3)	358 (4.0)	1,750 (2.6)	4,093 (4.0)
Descending colon	10,938 (6.0)	208 (5.4)	616 (7.0)	3,798 (5.6)	6,316 (6.2)
Sigmoid colon	50,234 (27.6)	997 (25.8)	2,457 (27.8)	18,992 (28.2)	27,788 (27.2)
Rectosigmoid junction	20,907 (11.5)	432 (11.2)	1,130 (12.8)	7,238 (10.7)	12,107 (11.9)
Rectum	36,848 (20.2)	1,513 (39.2)	2,294 (25.9)	15,526 (23.0)	17,515 (17.2)

a Early = localized; advanced = regional (with or without lymph node extension) or distant.

b Column percentages except for the Total row.

c Other race includes Asian/Pacific Islander, Native American, Asian/Indian, or Pakistani.

d US census tract neighborhood level socioeconomic status defined as the percentage of people living below poverty level as low (20%–100%), middle-low (10% to <20%), middle-high (5% to <10%), and highest (0% to <5%).

e Unknown smoking status refers to nonresponse or refused to respond.

Patients younger than 50 years were significantly more likely to present with advanced stage CRC compared with those aged 50 or older (AOR, 1.53; 95% CI, 1.47–1.60) (Table 2). Among patients younger than 50 years, black (AOR, 1.15; 95% CI, 1.03–1.29) and other race (AOR, 1.43; 95% CI, 1.10–1.84) had significantly higher odds of advanced stage CRC presentation compared with whites. Compared with the rectum, all anatomic sites showed significantly higher odds of advanced stage presentation in both age groups. For patients 50 years or older, Hispanics had significantly higher odds of advanced stage presentation compared non-Hispanics, although this association was not significant in those aged less than 50 years.

**Table 2:** Multivariable Logistic Regression Models Predicting Advanced Stage Colon Cancer^a^ Presentation at Diagnosis, Florida Cancer Data System, 1981–2013

Characteristic	All, AOR (95% CI)	Age Group	
		<50 years, AOR (95% CI)	≥ 50 years, AOR (95% CI)
**Age-group (reference, ≥50 years)**			
<50 years	1.53 (1.47–1.60)	NA	
**Sex (reference, male)**			
Female	1.00 (0.98–1.02)	0.88 (0.81–0.95)	1.01 (0.99–1.03)
**Race (reference, white)**			
Black	1.16 (1.12–1.21)	1.15 (1.03–1.29)	1.16 (1.12–1.21)
Other^b^	1.21 (1.10–1.34)	1.43 (1.10–1.84)	1.18 (1.06–1.31)
**Ethnicity (reference, non-Hispanic)**			
Hispanic	1.08 (1.05–1.12)	1.09 (0.97–1.21)	1.08 (1.04–1.12)
**Neighborhood socioeconomic status^c^ (reference, highest)**			
Lowest	1.10 (1.06–1.14)	1.16 (1.01–1.33)	1.09 (1.05–1.14)
Middle-low	1.06 (1.03–1.09)	1.16 (1.04–1.30)	1.05 (1.02–1.08)
Middle-high	0.99 (0.96–1.02)	1.12 (1.00–1.25)	0.98 (0.96–1.01)
**Smoking status (reference, never)**			
Current	1.26 (1.22–1.30)	1.19 (1.07–1.32)	1.26 (1.22–1.31)
Former	1.05 (1.02–1.08)	1.11 (0.98–1.25)	1.05 (1.02–1.08)
Unknown	0.94 (0.91–0.96)	1.00 (0.91–1.11)	0.93 (0.91–0.96)
**Anatomic site (reference, rectum)**			
Ascending colon	1.49 (1.44–1.53)	2.08 (1.81–2.39)	1.45 (1.41–1.50)
Hepatic flexure	1.59 (1.52–1.67)	1.78 (1.41–2.25)	1.57 (1.49–1.65)
Transverse colon	1.70 (1.63–1.76)	2.33 (1.96–2.78)	1.66 (1.59–1.72)
Splenic flexure	2.12 (2.00–2.24)	2.61 (2.06–3.33)	2.08 (1.95–2.20)
Descending colon	1.51 (1.45–1.58)	1.97 (1.66–2.34)	1.48 (1.41–1.55)
Sigmoid colon	1.33 (1.30–1.37)	1.66 (1.50–1.83)	1.31 (1.27–1.34)
Rectosigmoid junction	1.51 (1.46–1.56)	1.75 (1.54–1.99)	1.49 (1.43–1.54)

Abbreviations: AOR, adjusted odds ratio; CI, confidence interval; NA, not applicable.

a Regional (with or without lymph node extension) or distant.

b Other race includes Asian/Pacific Islander, Native American, Asian/Indian, or Pakistani.

c US census tract neighborhood level socioeconomic status defined as the percentage of people living below poverty level as low (20%–100%), middle-low (10% to <20%), middle-high (5% to <10%), and highest (0% to <5%).

## Discussion

Although patients aged less than 50 years are less likely to have CRC, our results demonstrate that these patients are significantly more likely to present with advanced stage CRC. Advanced stage CRC diagnosis is linked to significantly worse survival for all age groups (7). These results support past studies showing higher rates of advanced stage CRC presentation among those aged less than 50 years (2). Advanced stage CRC diagnosis may be attributable to misdiagnosis, genetic predisposition, or lack of knowledge regarding CRC symptoms (9). Consequently, recognizing and acknowledging risk factors for advanced stage CRC are necessary for earlier detection.

One of the most significant risk factors for advanced stage presentation among patients younger than 50 years was being black or other race. Higher rates of advanced stage presentation among blacks may be attributable to decreased awareness or patient attitudes toward CRC screening (10). These barriers prevent timely screening even in the presence of CRC symptoms. In addition, epidemiological studies have shown blacks develop CRC earlier than whites do (11). In terms of sex, women were significantly less likely to present with late-stage CRC in those younger than 50 years; however, there was no significant difference between men and women among those aged 50 or older. Low awareness and limited screening coupled with a predisposition for earlier CRC development warrant targeted public health efforts to raise awareness for CRC signs and symptoms as well as screening.

Being a patient younger than 50 years or being a former or current smoker was a significant predictor of having advanced CRC presentation. Although there is some debate regarding the role of tobacco smoking on CRC development, our results show that current and former smokers have significantly increased risk of advanced stage CRC presentation (12,13). Future studies are necessary to further discern the role of tobacco smoking in CRC development and progression.

In total, Hispanics had fewer cases of CRC compared with non-Hispanics. Studies have shown that Hispanics have both lower rates of CRC incidence and mortality (14). Our results show that Hispanics have significantly higher risk for advanced presentation of CRC among patients aged 50 or older, whereas the risk was not significantly increased among those aged less than 50 years. Pollack et al reported that Hispanics have lower rates of CRC screening, which may account for the increased rates of advanced CRC presentation in our results (15). The lower rates of screening among Hispanics may be attributable to limited health insurance. In addition, barriers to CRC screening in the Hispanic population include limited education, lower socioeconomic status, and cultural barriers (16,17). In terms of CRC screening among the Florida population, Hispanics report lower rates of fecal occult blood test (FOBT) testing and sigmoidoscopy or colonoscopy (18). Culturally appropriate public health interventions are necessary to increase CRC awareness and screening among the Hispanic population.

In terms of anatomic site, we found that there was a significantly lower risk of advanced stage CRC of the rectum across all ages, yet the rectum was the second most common site for tumor after behind the sigmoid colon. Siegel et al noted that rectal cancer incidence was increasing at a greater rate than colon cancer among patients aged less than 50 years (1). In our study, patients aged less than 50 years had significantly increased risk for advanced presentation for tumors arising outside of the rectum. This finding was also true for patients aged 50 or older, but the risks were higher in those younger than 50 years compared with those 50 or older across all locations. CRC presents differently depending on tumor location, and screening and awareness efforts should emphasize the different CRC presentations. Although data suggest that anatomic site does not significantly influence CRC presentation, anatomic location of CRC presentation may affect screening modality choice. Immunochemical testing such as FOBT has better sensitivity for detecting left-sided lesions compared with right-sided lesions (19).

This study is not without limitations. The FCDS database does not contain lifestyle habits such as dietary intake, which is significantly linked to CRC development (20). In addition, this cancer registry lacks genetic information and family history; both play a role in earlier CRC development. FCDS also does not include information about patients’ comorbidities such as diabetes and obesity, which could affect the risk and the stage of cancer.

Routine screening for individuals aged less than 50 years is not recommended unless the individuals are considered at high risk. However, multiple noninvasive CRC screening modalities such as FOBT have potential for noninvasive screening in high-risk populations (21). These noninvasive CRC screening modalities may offer an opportunity to increase CRC screening in groups with elevated risk factors for advanced stage presentation.

Our study highlights the increased risk for advanced stage CRC presentation in patients younger than 50 years compared with those aged 50 or older. With CRC incidence on the rise in younger US adults, raising awareness for CRC screening in high-risk groups is imperative to improve earlier detection. Increased awareness among health professionals is also necessary to address and to investigate potential signs of CRC in high-risk populations. Our results show that there are clear sociodemographic disparities in advanced stage CRC presentation. The increased risk for advanced stage CRC among patients younger than 50 years and select sociodemographic groups merits further investigation. Future efforts are warranted to further improve CRC screening and awareness among younger adults.

## Post-Test Information

To obtain credit, you should first read the journal article. After reading the article, you should be able to answer the following, related, multiple-choice questions. To complete the questions (with a minimum 75% passing score) and earn continuing medical education (CME) credit, please go to http://www.medscape.org/journal/pcd. Credit cannot be obtained for tests completed on paper, although you may use the worksheet below to keep a record of your answers.

You must be a registered user on http://www.medscape.org. If you are not registered on http://www.medscape.org, please click on the “Register” link on the right hand side of the website.

Only one answer is correct for each question. Once you successfully answer all post-test questions, you will be able to view and/or print your certificate. For questions regarding this activity, contact the accredited provider, CME@medscape.net. For technical assistance, contact CME@medscape.net. American Medical Association’s Physician’s Recognition Award (AMA PRA) credits are accepted in the US as evidence of participation in CME activities. For further information on this award, please go to https://www.ama-assn.org. The AMA has determined that physicians not licensed in the US who participate in this CME activity are eligible for *AMA PRA Category 1 Credits™*. Through agreements that the AMA has made with agencies in some countries, AMA PRA credit may be acceptable as evidence of participation in CME activities. If you are not licensed in the US, please complete the questions online, print the AMA PRA CME credit certificate, and present it to your national medical association for review.

## Post-Test Questions

### Study Title: Age-Specific Risk Factors for Advanced Stage Colorectal Cancer, 1981–2013

#### CME Questions

1. You are seeing a 45-year-old man whose sister (age 51 years) was just diagnosed with advanced colorectal cancer. The patient feels well and denies any other family history of colorectal cancer. He wants to know whether he should undergo screening for colorectal cancer. What can you tell him about the epidemiology of colorectal cancer in the United States?
  - The prevalence of colorectal cancer has risen steadily among adults during the past 2 decades
  - The prevalence of colorectal cancer has been rising particularly among adults younger than 50 years
  - Colorectal cancer is the third most commonly diagnosed cancer among women only
  - Colorectal cancer has become the most commonly diagnosed cancer among men
2. Which one of the following statements regarding cases of colorectal cancer diagnosed before age 50 years in the current study is most accurate?
  - Adults younger than 50 years made up 28% of the total number of cases of colorectal cancer
  - Most younger adults with colorectal cancer were women
  - The average age at the time of diagnosis of colorectal cancer younger adults was 42.5 years
  - Most cancers were located in the ascending colon among younger adults
3. Which one of the following variables was most protective against the risk for advanced colorectal cancer among adults younger than 50 years in the current study?
  - Female sex
  - Current smoking
  - Middle income level
  - Hispanic ethnicity
4. Which one of the following statements regarding other risk factors for advanced colorectal cancer in the current study is most accurate?
  - Age older than 50 years was a significant risk factor for advanced cancer
  - Black race was associated with a higher risk for cancer among younger and older adults
  - Smoking did not influence the risk for advanced cancer
  - The ascending colon was the anatomic site associated with the lowest rate of advanced cancer

## References

[1] Siegel RL , Fedewa SA , Anderson WF , Miller KD , Ma J , Rosenberg PS , Colorectal cancer incidence patterns in the United States, 1974–2013. J Natl Cancer Inst 2017;109(8):djw322. 10.1093/jnci/djw322 28376186 PMC6059239

[2] Fairley TL , Cardinez CJ , Martin J , Alley L , Friedman C , Edwards B , Colorectal cancer in U.S. adults younger than 50 years of age, 1998–2001. Cancer 2006;107(5, Suppl):1153–61. 10.1002/cncr.22012 16862554

[3] Siegel RL , Miller KD , Fedewa SA , Ahnen DJ , Meester RGS , Barzi A , Colorectal cancer statistics, 2017. CA Cancer J Clin 2017;67(3):177–93. 10.3322/caac.21395 28248415

[4] Siegel RL , Miller KD , Jemal A . Cancer statistics, 2016. CA Cancer J Clin 2016;66(1):7–30. 10.3322/caac.21332 26742998

[5] United States Cancer Statistics. 1999–2014 Incidence and Mortality Web-based Report. U.S. Department of Health and Human Services, Centers for Disease Control and Prevention and National Cancer Institute. 2017. https://nccd.cdc.gov/USCS/statevsnational.aspx?Year=2014&Variable1=Florida. Accessed May 17, 2017.

[6] Mokdad AH , Dwyer-Lindgren L , Fitzmaurice C , Stubbs RW , Bertozzi-Villa A , Morozoff C , Trends and patterns of disparities in cancer mortality among US counties, 1980–2014. JAMA 2017;317(4):388–406. 10.1001/jama.2016.20324 28118455 PMC5617139

[7] Tannenbaum SL , Hernandez M , Zheng DD , Sussman DA , Lee DJ . Individual- and neighborhood-level predictors of mortality in Florida colorectal cancer patients. PLoS One 2014;9(8):e106322. 10.1371/journal.pone.0106322 25170910 PMC4149543

[8] US Preventive Services Task Force, Bibbins-Domingo K , Grossman DC , Curry SJ , Davidson KW , Epling JW Jr , Screening for colorectal cancer: US Preventive Services Task Force recommendation statement. JAMA 2016;315(23):2564–75. Errata in JAMA 2016;316(5):545 and JAMA 2017;317(21):2239. 10.1001/jama.2016.5989 27304597

[9] O’Connell JB , Maggard MA , Liu JH , Etzioni DA , Livingston EH , Ko CY . Do young colon cancer patients have worse outcomes? World J Surg 2004;28(6):558–62. 10.1007/s00268-004-7306-7 15366745

[10] Greiner KA , Born W , Nollen N , Ahluwalia JS . Knowledge and perceptions of colorectal cancer screening among urban African Americans. J Gen Intern Med 2005;20(11):977–83. 10.1007/s11606-005-0244-8 16307620 PMC1490251

[11] Agrawal S , Bhupinderjit A , Bhutani MS , Boardman L , Nguyen C , Romero Y , Colorectal cancer in African Americans. Am J Gastroenterol 2005;100(3):515–23, discussion 514. Erratum in Am J Gastroenterol 2005;100(6):1432. 10.1111/j.1572-0241.2005.41829.x 15743345

[12] Giovannucci E . An updated review of the epidemiological evidence that cigarette smoking increases risk of colorectal cancer. Cancer Epidemiol Biomarkers Prev 2001;10(7):725–31. 11440957

[13] Parajuli R , Bjerkaas E , Tverdal A , Selmer R , Le Marchand L , Weiderpass E , The increased risk of colon cancer due to cigarette smoking may be greater in women than men. Cancer Epidemiol Biomarkers Prev 2013;22(5):862–71. 10.1158/1055-9965.EPI-12-1351 23632818

[14] O’Brien K , Cokkinides V , Jemal A , Cardinez CJ , Murray T , Samuels A , Cancer statistics for Hispanics, 2003. CA Cancer J Clin 2003;53(4):208–26. Erratum in CA Cancer J Clin 2003;53(5):314. 10.3322/canjclin.53.4.208 12924775

[15] Pollack LA , Blackman DK , Wilson KM , Seeff LC , Nadel MR . Colorectal cancer test use among Hispanic and non-Hispanic U.S. populations. Prev Chronic Dis 2006;3(2):A50. 16539791 PMC1563962

[16] Vidal L , LeBlanc WG , McCollister KE , Arheart KL , Chung-Bridges K , Christ S , Cancer screening in US workers. Am J Public Health 2009;99(1):59–65. 10.2105/AJPH.2008.135699 19008502 PMC2636597

[17] Stefanidis D , Pollock BH , Miranda J , Wong A , Sharkey FE , Rousseau DL , Colorectal cancer in Hispanics: a population at risk for earlier onset, advanced disease, and decreased survival. Am J Clin Oncol 2006;29(2):123–6. 10.1097/01.coc.0000199918.31226.f8 16601428

[18] Centers for Disease Control and Prevention, National Center for Chronic Disease Prevention and Health Promotion, Division of Population Health. BRFSS prevalence and trends data. 2015. https://www.cdc.gov/brfss/brfssprevalence/. Accessed May 17, 2017.

[19] Haug U , Kuntz KM , Knudsen AB , Hundt S , Brenner H . Sensitivity of immunochemical faecal occult blood testing for detecting left- vs right-sided colorectal neoplasia. Br J Cancer 2011;104(11):1779–85. 10.1038/bjc.2011.160 21559011 PMC3111170

[20] Baena R , Salinas P . Diet and colorectal cancer. Maturitas 2015;80(3):258–64. 10.1016/j.maturitas.2014.12.017 25619144

[21] Smith RA , Cokkinides V , Brawley OW . Cancer screening in the United States, 2008: a review of current American Cancer Society guidelines and cancer screening issues. CA Cancer J Clin 2008;58(3):161–79. 10.3322/CA.2007.0017 18443206

